# Heterophase Polymorph of TiO_2_ (Anatase, Rutile, Brookite, TiO_2_ (B)) for Efficient Photocatalyst: Fabrication and Activity

**DOI:** 10.3390/nano13040704

**Published:** 2023-02-12

**Authors:** Diana Rakhmawaty Eddy, Muhamad Diki Permana, Lintang Kumoro Sakti, Geometry Amal Nur Sheha, Solihudin, Sahrul Hidayat, Takahiro Takei, Nobuhiro Kumada, Iman Rahayu

**Affiliations:** 1 Department of Chemistry, Faculty of Mathematics and Natural Sciences, Universitas Padjadjaran, Sumedang 45363, West Java, Indonesia; 2 Integrated Graduate School of Medicine, Engineering, and Agricultural Sciences, University of Yamanashi, Kofu 400-8511, Japan; 3 Center for Crystal Science and Technology, University of Yamanashi, Kofu 400-8511, Japan; 4 Department of Physics, Faculty of Mathematics and Natural Sciences, Universitas Padjadjaran, Sumedang 45363, West Java, Indonesia

**Keywords:** titanium dioxide, heterophase, polymorph, anatase, rutile, photocatalysis

## Abstract

TiO_2_ exists naturally in three crystalline forms: Anatase, rutile, brookite, and TiO_2_ (B). These polymorphs exhibit different properties and consequently different photocatalytic performances. This paper aims to clarify the differences between titanium dioxide polymorphs, and the differences in homophase, biphase, and triphase properties in various photocatalytic applications. However, homophase TiO_2_ has various disadvantages such as high recombination rates and low adsorption capacity. Meanwhile, TiO_2_ heterophase can effectively stimulate electron transfer from one phase to another causing superior photocatalytic performance. Various studies have reported the biphase of polymorph TiO_2_ such as anatase/rutile, anatase/brookite, rutile/brookite, and anatase/TiO_2_ (B). In addition, this paper also presents the triphase of the TiO_2_ polymorph. This review is mainly focused on information regarding the heterophase of the TiO_2_ polymorph, fabrication of heterophase synthesis, and its application as a photocatalyst.

## 1. Introduction

Titanium dioxide, also known as titania, is a naturally occurring transition metal oxide of titanium with the chemical formula TiO_2_ [1]. Titanium dioxide is the most effective and efficient semiconductor material as a photocatalyst, has good stability, and has high ultraviolet absorption compared to other materials [2,3,4,5,6]. In addition, TiO_2_ is a commercially available materials for applications in various fields due to its wide availability, biocompatibility, and non-toxicity [7,8,9]. TiO_2_ has a white colour which is used as a pigment in paints, printing inks, plastics, ceramics, and cosmetics [10,11,12,13]. TiO_2_ is the most effective and efficient semiconductor material, and has also been reported in the photoelectrical devices [14,15]. TiO_2_ also has electronic and optical properties that can be utilized in the field of photocatalysts [16], self-cleaning materials [17], and solar cells [18].

Titanium dioxide has many applications as a photocatalyst, for example, in hydrogen production [19,20,21,22], degradation of organic compounds [23,24,25,26,27], remediation of metal ions [28,29,30], and synthesis of organic compounds [31,32,33,34]. Most of the research on TiO_2_ has been carried out to increase its photocatalytic efficiency [35]. Many studies have also been devoted to the synthesis of various forms of nanomaterials [36,37,38], engineered with doping [39,40,41,42] or composites [43,44,45].

TiO_2_ exists naturally in three crystalline forms, anatase, rutile, and brookite [46,47,48,49]. Besides that, there is another polymorph, namely TiO_2_ (B) [50,51]. These polymorphs exhibit different properties and, consequently, different photocatalytic performances [52]. In general, many studies have stated that anatase has the best photocatalytic activity [53]. However, in some cases, rutile is more active as a photocatalyst [54]. In addition, the binary mixture of TiO_2_ polymorphs showed a significant increase in the rate of catalytic activity for several reactions. Among these phases, the binary phase of anatase and rutile is the most studied phase [55,56].

The main drawback of photocatalyst application is the high recombination rate of electrons and holes which will reduce the quantum efficiency and decrease the photocatalytic activity [57,58]. One way to overcome this problem is to use multiphase TiO_2_ because it exhibits higher photocatalytic activity than single-phase due to the possible charge transfer generated between different TiO_2_ polymorphs (with different levels of electronic bands), leading to an effective separation of charge carriers thereby preventing recombination of electrons and holes [59,60].

The TiO_2_ heterophase can effectively stimulate electron transfer from one phase to another cause superior photocatalytic performance [61,62]. For example, TiO_2_ P25 Degussa, which consists of ∼20% rutile and ∼80% anatase, is an excellent photocatalyst [63]. Several studies have succeeded in synthesizing TiO_2_ heterophase which shows better photocatalytic abilities than pure anatase, namely, anatase/rutile [64] and anatase/brookite [65]. Growing research efforts have recently been devoted to new TiO_2_ heterophase composites including anatase/TiO_2_ (B) [66,67] and rutile/brookite [68], as well as the three-phase anatase/rutile/brookite [69] and anatase/rutile/TiO_2_ (B) [70]. Wang et al. [71] reported a novel approach to fabricate heterophase anatase/TiO_2_ (B) in which heterophase can be obtained at 550 °C. The results show that although in many cases the photocatalytic activity of TiO_2_ (B) is lower than that of anatase, a suitable composition between anatase and TiO_2_ (B) will lead to increased activity [72].

As described in detail by other researchers previously, the most obvious advantage of the multiphase photocatalyst is that it can promote electron–hole separation, thereby enhancing the photocatalytic activity of materials. Therefore, in the photocatalytic system, it is very important to synthesize a photocatalyst with a multiphase structure and high degradation efficiency. This review mainly addresses reports related to TiO_2_ heterophase over the past 10 years, in which a wider field of research has been reported. This work aims to clarify the differences between titanium dioxide polymorphs, and the differences in homophase and heterophase (biphase and triphase) properties in various photocatalytic applications. This review includes information on TiO_2_ polymorph heterophase, the fabrication of synthetic heterophase, and their applications as photocatalysts. This review ends with conclusions and perspectives, which may stimulate further development of the utility of TiO_2_ heterophase. Review articles covering heterophase with thorough explanations have not been reported. Other reviews have focused on anatase and rutile [73,74,75] with other composite or doped materials [76,77,78,79,80].

## 2. Homophase TiO_2_

### 2.1. Photocatalysis Mechanism of TiO_2_

TiO_2_ is a semiconductor material, which is a substance that lies between conductors (such as metals) and insulators (such as ceramics) [81]. In semiconductor, the distance between the position of the valence band (VB) and the conduction band (CB) determine the ability of the semiconductor material in the light absorption process and its oxidation-reduction ability [82,83,84,85]. In general, the photocatalytic reaction of TiO_2_ includes several basic processes, such as the formation of charge carriers, separation, relaxation, capture, transfer, recombination, and transport [86,87,88]. This process must be thoroughly observed to understand the workings of TiO_2_ photocatalysts and is useful for the development of new photocatalysts [89,90].

**Figure 1:**
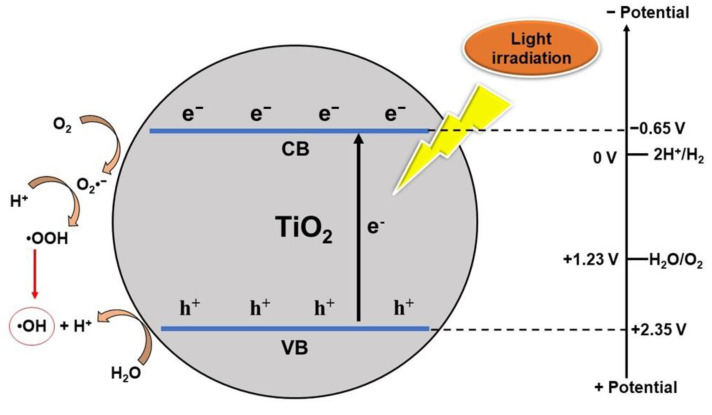
Schematic diagram of TiO_2_ photocatalytic principle.

In general, the mechanism of photocatalysis using TiO_2_ is illustrated in Figure 1. When TiO_2_ material is subjected to photon irradiation with an energy greater than the band gap of TiO_2_, the electrons in VB will be excited to CB resulting in holes in VB [91,92,93,94,95]. The process of photoexcitation of pairs of electrons (*e*^−^) and holes (*h*^+^) will participate in redox reactions with adsorbed species which will form superoxide radical anions (•O^2−^) and hydroxyl radicals (•OH), respectively, which will play a role in the degradation of organic pollutants in water [96,97,98,99]. Only photons with energies greater than the band-gap energy can excite electrons and drive reactions to occur. The activation of TiO_2_ by UV light can be written as Equations (1)–(3) [100,101,102]. In this reaction, *h*^+^ and *e*^−^ are strong oxidizing and reducing agents.

TiO_2_ + *hν* → *e*^−^ + *h*^+^ (1)

*e*^−^ + O_2_ → O_2_•^−^ (2)

*h*^+^ + H_2_O → •OH + H^+^ (3)

Reactive oxygen species (ROS) produced during the photocatalytic process vary greatly, mainly the ROS that is formed is the superoxide radical O_2_•^−^ and the hydroxyl radical •OH. However, other species such as •OOH and H_2_O_2_ may be formed through further oxidation processes, dimerization, or disproportionation [103,104,105].

### 2.2. Phase of TiO_2_

**Figure 2:**
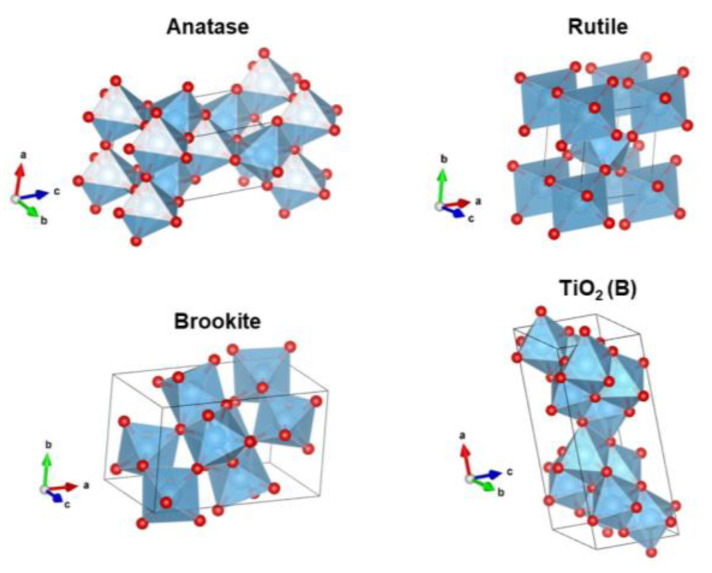
Crystal structure of TiO_2_ anatase, rutile, brookite, and TiO_2_ (B).

TiO_2_ is widely available in nature as rutile, anatase, and brookite polymorphs; these three types are octahedral TiO_6_ with different distortions [106,107]. The structures of anatase, rutile, and brookite are shown in Figure 2 [108]. While rutile is a stable phase, both anatase and brookite are metastable phases. In addition, this brookite is difficult to synthesize, so it is rarely studied [109,110,111,112]. There is another polymorph found from TiO_2_, namely TiO_2_ (B) [113,114,115]. The characteristics of the Ti-O bond determine the differences in the structural and electronic properties of the different TiO_2_ phases [116]. Table 1 shows the different properties of the TiO_2_ polymorph [117].

**Figure 3:**
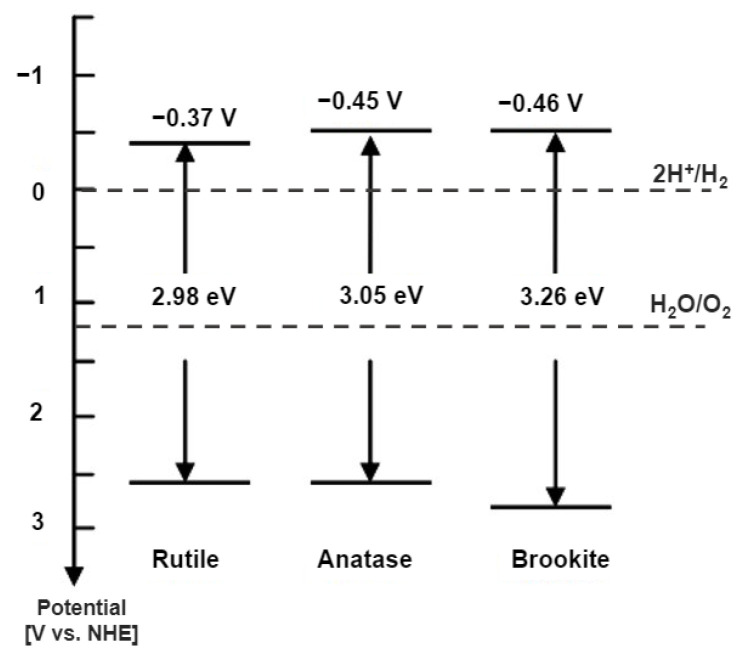
Bandgap energies, VB, and CB for anatase, rutile, and brookite on the potential scale (V) versus the normal hydrogen electrode (NHE).

In general, anatase showed higher photocatalytic activity than rutile. However, the reason for the difference in photocatalytic activity between these phases is still being debated [118,119]. Zhang et al. [120] showed that anatase is a semiconductor with an indirect band gap, while rutile and brookite are included in the category of direct band gap semiconductors. In addition, photocatalytic effects such as organic pollutant decomposition will be successful if the semiconductor material has a band gap energy of redox potential at the hole that is VB positive enough to produce hydroxyl radicals and electrons in CB, which must be negative enough to produce superoxide radicals (E_0_(H_2_O/•OH) = 2.8 V vs. NHE) and (E_0_(O_2_/O_2_^•−^) = −0.28 V vs. NHE). Figure 3 shows the band gap energies of anatase, rutile, and brookite TiO_2_ [121,122,123].

#### 2.2.1. Anatase

Anatase polymorph has better photocatalytic activity than other TiO_2_ polymorphs [124,125,126,127,128]. Anatase has a larger band gap than rutile. This increases the oxidizing ability and facilitates electron transfer. Recent results show that the anatase has a lower conduction band than the rutile [129]. Moreover, the indirect band gap of the anatase form is smaller than the direct band gap. Thus, the indirect band gap is used for the photocatalysis process of the anatase form. For rutile, on the other hand, the fundamental band gap of indirect band gap is very similar to the direct band gap. Thus, the rutile type tends to excite electrons in a direct band gap. Indirect band gap materials generally exhibit a longer charge carrier lifetime compared to direct band gap materials [130,131,132,133], thus making anatase have better activity in most cases compared to rutile and brookite.

Anatase has a much wider specific surface area, so it has more active sites than rutile. Anatase also has a higher oxygen vacancy concentration than rutile, which is the reason for anatase’s higher charge separation efficiency [134]. The adsorption affinity of anatase for organic compounds is higher than that of rutile, and anatase shows a lower recombination rate than that of rutile because of its higher hole-trapping rate [135]. The adsorption affinity is defined as the equilibrium ratio of the solid-phase concentration to the liquid-phase solute concentration, also known as adsorption equilibrium constant [136]. However, even though it has better photocatalytic activity, the single anatase phase has low thermodynamic stability when compared to the rutile phase, so it can only be synthesized in several types of synthesis [137,138].

**Figure 4:**
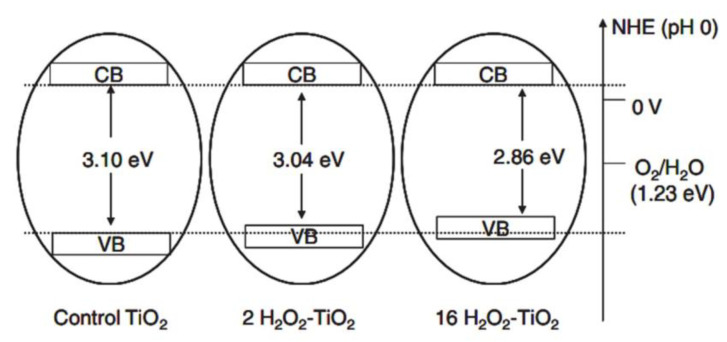
Mechanism of band gap narrowing for peroxo-titania (anatase) complex. Reproduced with permission from [140]. Copyright John Wiley and Sons, 2011.

Bubacz et al. [139] synthesized anatase TiO_2_ with TiOSO_4_ precursor in ammonia water as a phenol degradation and azo dye. The decreased concentrations of the dye and phenol was measured by UV/VIS spectroscopy and a TOC analyzer. Polycrystalline of anatase was produced with a crystalline size of 12.7–13.0 nm and a particle size of 195.7 nm. The material produces satisfactory activity with an optimum pH of 6.5. In addition, Etacheri et al. [140] synthesized anatase with a band gap that can be reduced (without doping) and can works in visible light. This is achieved by generating oxygen in situ via the thermal decomposition of the peroxo–titania complex (H_2_O_2_-TiO_2_). The increased strength of the Ti–O–Ti bond and the maximum shift of VB are responsible for the stability in high temperature and visible light activity (Figure 4).

In the report of Lv et al. [141], anatase is modified using fluorine as a shape guide for surface modification. This modification produces various morphological forms such as nanotubes, titania sheets with {001} high-energy facets, and hollow spheres. This form results in increased diverse photocatalytic activity. In this regard, regarding the effect of fluoride on the structure and photocatalytic activity of TiO_2_, many questions remain open. In another report, Wang et al. [142] reported the differences in the photocatalytic activity ability between anatase and rutile mesopores. Mesoporous anatase results in long-lived electron generation and allows for efficient electron transfer. Meanwhile, mesoporous rutile is substantially less efficient and more reversible than anatase.

#### 2.2.2. Rutile

The rutile phase becomes an attractive polymorph TiO_2_ for photocatalytic applications because it can absorb UV light near the visible region. The band gap of rutile is 3.0 eV, lower than that of anatase (3.2 eV). However, the rutile phase has not been widely studied as a photocatalyst because of its lower photocatalytic activity than anatase. Its low specific surface area, fast recombination, and the positive location of its minimum conduction band make it have a poorer reducing ability than anatase [143,144,145,146,147].

Rutile is the most stable TiO_2_ polymorph and is easily produced at high temperatures. However, rutile has rarely been studied as a photocatalyst for the oxidation of dyes/organic compounds, due to poor oxygen uptake due to the lower valence band position compared to anatase [148]. In the research by Wang et al. [149], rutile was successfully synthesized using low temperatures. Pure rutile was synthesized by hydrolysis of an aqueous TiCl_4_ ethanol solution at 50 °C. The results show that rutile synthesized at low temperatures exhibits much higher photocatalytic activity than commercial P25 photocatalysts in the degradation of rhodamine B. Applications of rutile photocatalysis include water separation. In contrast to anatase, rutile allows preferential water oxidation, which is useful for the construction of Z-scheme water separation systems [150]. In addition, in another study, rutile nanoparticles with large specific surface areas and abundant oxygen vacancies were designed for photocatalytic nitrogen fixation [151]. Meanwhile, in a study conducted by Yurdakal et al. [152], rutile was used for the selective oxidation of aromatic alcohols to aldehydes in an aqueous suspension.

The photocatalytic abilities of rutile and anatase were compared by Jung and Kim [153] to stearic acid and measured the rate of decrease in the integrated absorbance of the ensemble of the C−H stretching vibrations using FTIR spectroscopy. The results show that although rutile nanoparticles have a higher specific surface area than anatase nanoparticles, the photocatalytic properties of rutile nanoparticles are much lower than those of anatase nanoparticles. This is attributed to the intrinsic radiative recombination of photogenerated electrons and holes in rutile. However, other studies have shown that the presence of the vacancy-oxygen defect (VO) in rutile can significantly increase the ability of photocatalytic activity. VO is known to suppress the charge recombination process [154]. Although in many studies anatase shows higher activity, in some cases, rutile can be superior [155]. For example, the research by Zhang et al. [156], compared the photocatalytic activity of anatase, rutile, and brookite for Rhodamine B (RhB) degradation. The results show that rutile provides a faster photocatalytic reaction rate than anatase for the same particle size and specific surface area.

#### 2.2.3. Brookite

Brookite has an orthorhombic crystal structure with the *Pbca* space group [157]. Brookite will turn into rutile at high temperatures. This conversion can occur directly to rutile or through the formation of anatase first. This depends on several factors such as crystallite size, size distribution, and crystallite contact area. [158]. For crystal sizes larger than 11 nm, brookite is more stable than anatase, while for sizes larger than 35 nm, rutile is the most stable phase [159]. Xie et al. [160] synthesized pure-phase brookite by hydrothermal method using Ti(SO_4_)_2_ as the precursor. Phase formation is achieved by hydrothermal treatment at 180 °C [161]. Kandiel et al. [162] observed that a direct transformation of anatase and brookite to rutile was observed, while in the case of anatase–brookite mixture, anatase changed first to brookite and then to rutile.

The photocatalytic activity of brookite was studied by Khan et al. and its activity is highly dependent on the level of disability. The results of the DFT calculations show that the Ti^4+^ defect is the main defect in increasing the photocatalytic activity of brookite [163,164]. One of the main reasons for the different catalytic performances of the TiO_2_ polymorph is the depth of charge carrier trapping. Electrons in brookite are trapped in shallow traps and not photogeneration, which reduces the amount of free electron, but on the other hand, this extends the life span of hole [165].

Bellardita et al. calculated the absolute crystallinity of brookite. This value was calculated from the ratio between the full width at half maximum (FWHM) intensity peak XRD pattern (121) of brookite and peak (111) of CaF_2_ as an internal standard. The results showed that crystallinity had a positive effect on the oxidation of 4-nitrophenol and 4-methoxybenzyl alcohol [166]. Choi et al. [167] synthesized pure brookite TiO_2_ films as a photocatalyst. The brookite phase was synthesized on titanium foil using a hydrothermal reaction. The results show that the brookite phase exhibits significantly higher photoactivity among the TiO_2_ polymorphs, despite its smaller specific surface area compared to anatase [168]. Tetramethylammonium (TMA) can be degraded in pure anatase and brookite phases, but not in rutile, where TMA concentration was measured by ion chromatograph [167].

#### 2.2.4. TiO_2_ (B)

TiO_2_ (B) is a polymorph of titanium dioxide discovered in 1980 which was prepared from the hydrolysis of K_2_Ti_4_O_9_ followed by heating at 500 °C. TiO_2_ (B) has a host covalent framework of Na_x_TiO_2_ bronze [169]. In 2004, Armstrong et al. [115] successfully prepared TiO_2_ (B) nanowires. The synthesis was carried out involving a hydrothermal reaction between NaOH and TiO_2_, followed by acid washing and heating at 400 °C. The synthesized TiO_2_ (B) has the potential to be a material in lithium-ion batteries because it has the advantage of having a relatively open structure. Therefore, it is an excellent host for Li intercalation [170,171,172,173]. TiO_2_ (B) nanoparticles were also developed with various sizes. The small particle size causes the dimensionality reduction to increase the amount of Li and hence the charge that can be stored [174].

The use of TiO_2_ (B) as a photocatalyst was carried out by Chakraborty et al. [175]. The results show that H_2_Ti_5_O_11_.H_2_O is a prerequisite for the formation of TiO_2_ (B) phase. The photocatalytic activity of TiO_2_ (B) showed 1.35 and 1.95 times higher in decomposing 4-chlorophenol than Degussa P25 and 25 nm sized anatase nanoparticle. This is due to the high crystallinity of TiO_2_ (B). In addition, in other studies, TiO_2_ (B) doped with Ti^3+^ provided increased photocatalytic performance in rhodamine B (RhB) decomposition, which was measured using a UV spectrophotometer and hydrogen evolution, which was measured using gas chromatography. The RhB degradation rate is ∼6.9 and the hydrogen evolution rate is 26.6 times higher compared to titanium dioxide nanoparticles [176].

### 2.3. Disadvantages of TiO_2_ Homophase Photocatalysis

**Figure 5:**
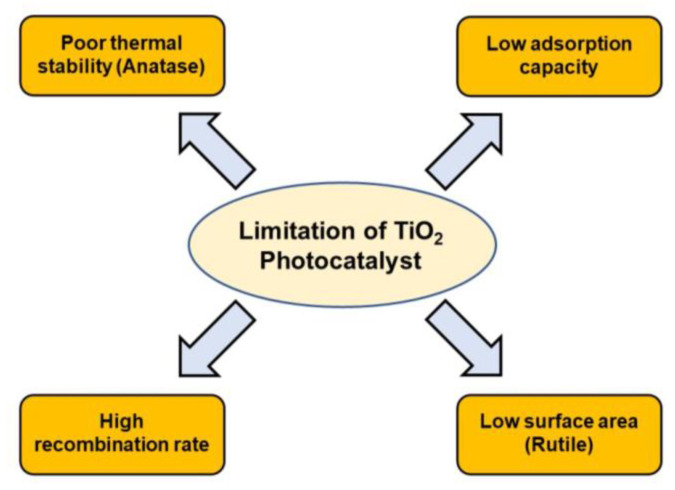
Limitations of TiO_2_ as a photocatalyst material.

TiO_2_ has great potential as a photocatalyst for the degradation of organic pollutants and microorganisms. However, in practice, there are many limitations, namely, high h^+^ and *e*^−^ recombination rates, low adsorption capacity, and low thermal stability of the anatase TiO_2_ phase (Figure 5). TiO_2_ has a wide band gap of 3.0−3.2 eV, meaning this photocatalyst mainly absorbs ultraviolet photons, while indoor lamps only emit visible light photons [177,178]. This causes the use of indoor photocatalysts such as disinfection to be very limited because they require UVA irradiation. Strategies that can be implemented to overcome this challenge include adding metals (Cu, Ag, Eu, Fe, Zn, and La) [179,180,181,182], adding non-metallic dopants (N, F, S, and C) [183,184,185,186], or the use of the photon induction method [187].

The crystallite size and phase of TiO_2_ are aspects that affect the photonic efficiency and recombination dynamics in the photocatalytic process. Samples with a single anatase phase showed a high percentage of recombination due to the influence of crystallographic and microstructural parameters [188]. Meanwhile, samples with a mixed phase, such as anatase/rutile, have a more prominent crystallography and microstructure between anatase (011) and (110) rutile, which causes a decrease in the recombination rate due to the presence of hole and electron transfers [189]. Meanwhile, in the single rutile phase, there is a decrease in the formation rate of •OH and *e*^−^ due to the less efficient formation of •OH species during the oxidation of water on the rutile particles, as well as the shorter lifetime and lower reactivity of *e*^−^ in the single rutile phase. This phenomenon indicates that the photocatalytic synergy associated with anatase/rutile mixtures is not always present, but depends on the relationship between the fermi level of the anatase and rutile particles and the characteristics of the particles [190]. Photocatalytic junctions, especially type-II heterojunctions between semiconductors, are considered a potential pathway to improve photocatalytic performance [191]. The homojunction heterophase that exploits polymorphism has potential advantages. It has homogeneous components and nearly perfect lattice matching, and they can conduct efficient charge transfer at the interface [192].

## 3. Heterophase TiO_2_ in Photocatalysts

### 3.1. Photocatalysis Mechanism of TiO_2_ Heterophase

**Figure 6:**
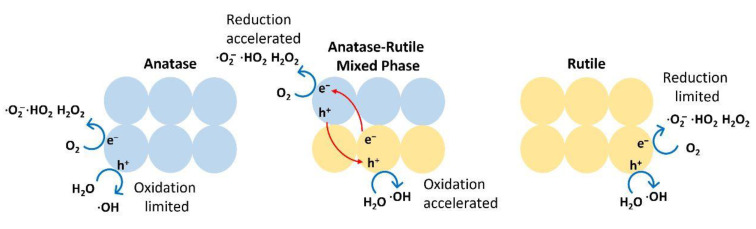
Mechanism of photocatalysis in anatase, rutile, and anatase–rutile heterophase.

TiO_2_ with mixed phases such as anatase and rutile showed better photocatalytic activity than TiO_2_ with 100% anatase phase. This increase in activity generally comes from the interaction between the two forms; the interaction reduces recombination due to a bulk structure of anatase/rutile. This mixed phase has an interfacial electron trapping site that enhances the photocatalytic activity of TiO_2_ [193]. Meanwhile, in the anatase structure, the oxidation process is limited, while in contrast to the rutile structure, the reduction process is limited. Thus, when it is TiO_2_ with the mixed phase, the oxidation-reduction process can be accelerated [194]. This is illustrated in Figure 6. When different phases of TiO_2_ are mixed, the rate of recombination is reduced, the lighting efficiency is increased, and the energy band gap can be activated by light with energies lower than UV [135]. Due to its high activity, P25 Degussa, a commercial TiO_2_, is frequently used as a benchmark in photocatalytic processes. It has a mixed phase of anatase and rutile with a ratio of 80% and 20%, respectively [195].

**Figure 7:**
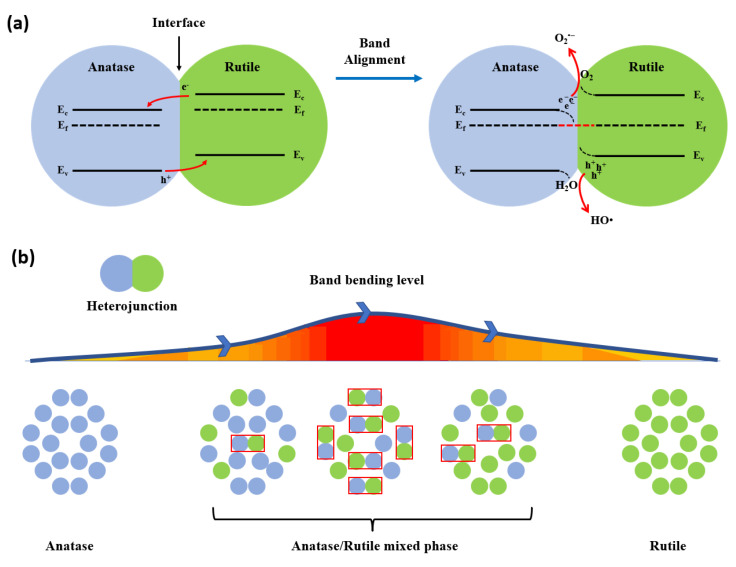
Heterojunction of mixed-phase TiO_2_ nanoparticles: (**a**) alignment of the conduction band and valence band between the rutile and anatase phases, which causes the separation of electron holes at the heterojunction; and (**b**) heterojunction density variations during the transformation of the anatase phase to rutile.

At semiconductor heterojunctions, energy bands of two different materials come together, leading to an interaction. Both band structures are positioned discontinuously from each other, causing them to align close to the interface [196]. This alignment is caused by the discontinuous band structures of the semiconductors when compared to each other and the interaction of the two surfaces at the interface [197]. In research by Scanlon et al. [198], there are signs that when there is contact, the valence band energy and conduction band energy are higher in the rutile phase than in the anatase phase. Electrons will go to the anatase because of the lower conduction band minimum energy, and holes will move to the rutile because of the higher valence band maximum energy, according to the alignment of the bending energy bands at the anatase/rutile interface. Then, the holes in the valence band will react with water to generate hydroxyl radicals, while the electrons in the conduction band will concurrently react with oxygen to generate superoxide anions [199,200]. Figure 7 shows that the energy band changes at the interface of the anatase/rutile heterojunctions can lead to electron–hole separation and band bending until the Fermi levels of the anatase and rutile are aligned, and the density of the heterojunction and the toxicity of the nanoparticles can be determined by the degree of the band bending. The degree of band bending can be used to experimentally show that the heterojunction density of the TiO_2_ mixed phase is theoretically proportional to the quantity of electron–holes at the heterojunction interface. Heterojunction density is the local density of state and charge density for heterojunction semiconductor (usually composites), which is calculated using computational software such as the Vienna ab initio simulation package (VASP) and Cambridge Sequential Total Energy Package (CASTEP) [201]. Furthermore, the ROS generated during the photocatalytic process and its potential in the oxidation process can be estimated by the band bending value [202]. Band bending value or degree of band bending is proportional to the amount of accumulated electrons or holes that depend on the density of heterojunction; this value can reflect the density of heterojunction in mixed-phase TiO_2_ [203].

### 3.2. Computational Study of Heterophase TiO_2_

**Figure 8:**
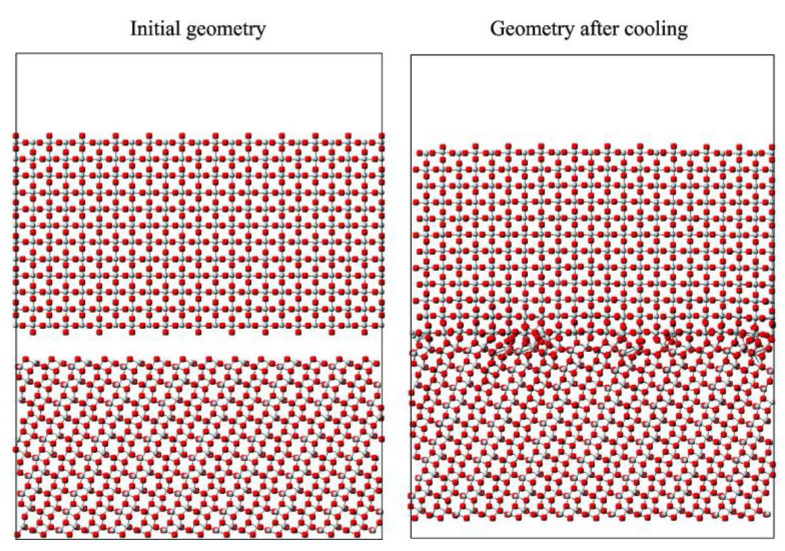
Interface formation between rutile (110) and anatase (101) before and after the cooling steps. Reproduced with permission from [206]. Copyright American Chemical Society, 2007.

Surface structures and interfacial sections are very important for photocatalytic processes because these points are considered as the focus of transfer and trapping of charge carrier species [204,205]. The possible arrangement at the interfacial point between anatase and rutile was analyzed by stacking the atomic layers of the anatase (011) plane and the (110) rutile plane [189]. In a study by Denkins et al. [206], two plates were prepared, one anatase and one rutile, each 30 Å thick, separated from each other by 5 Å. Then, MD simulations were carried out for 400 ps at 1300 K. An illustration of the initial and final structures is shown in Figure 8. As shown, a vacuum distance of about 25 Å is present to minimize interactions between non-interfacial surfaces.

**Figure 9:**
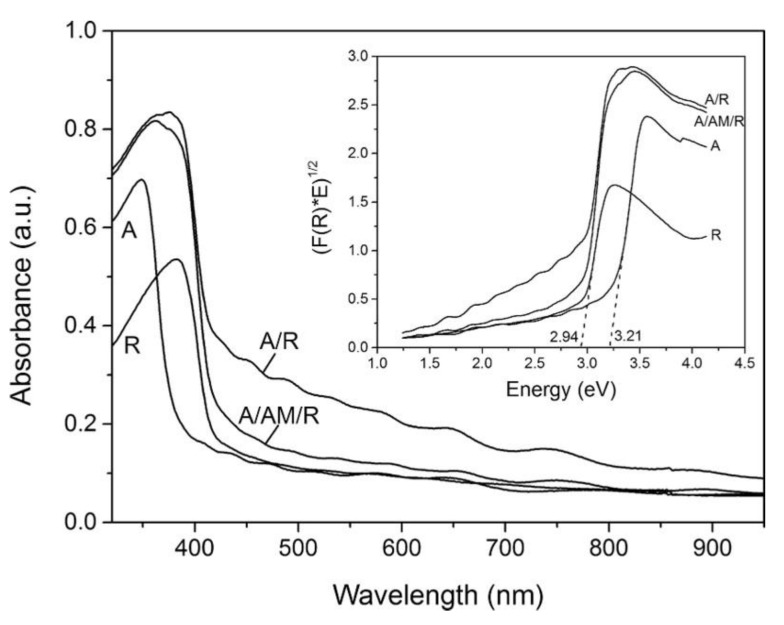
Absorption spectra of anatase (A), rutile I, multilayer films before (A/Am/R) and after (A/R) heat treatment at 500 °C for 10 h. Inset: Tauc plots showing approximate bandgap energy values of the A,R crystalline phases. Reproduced with permission from [209]. Copyright John Wiley and Sons, 2014.

The band gap of the structure of the TiO_2_ mixture measured using HSE06 is around 3.0 eV which comes from the alignment band between the anatase (3.5 eV) and rutile (3.2 eV) phases [207]. The quantum size effect is responsible for the distinction in the band-gap values (approximately 0.2 eV) of each phase. The energy level of the Ti d orbitals can be adjusted by various coordination configurations, which makes this plausible. Additionally, a significant component in determining the photocatalytic activity linked to geometric and electrical structures is the absorption of photons from a semiconductor photocatalyst [208]. There is no significant change for the absorption curves of the mixed structure compared to the individual components of anatase and rutile. It shows that there is little orbital overlap and little electronic transition between rutile and anatase, and that direct electronic infusion of rutile into anatase hardly ever occurs during the excitation process. The structural changes that form the interface have no significant effect on the absorption of light by mixed-phase TiO_2_ and the optical absorption of this structure occurs in the anatase and rutile phases, respectively [205]. In another study, anatase/rutile (A/R) and interlayer amorphous (Am) films A/Am/R were synthesized using atmospheric pressure chemical vapor deposition (APCVD) to produce the absorption and bandgap shown in Figure 9. The gap energy of the band for the anatase/rutile heterojunction (2.78 eV) appears red-shifted from the pure component [209].

### 3.3. Fabrication Methods of Heterophase TiO_2_

#### 3.3.1. Sol–Gel Method

**Figure 10:**
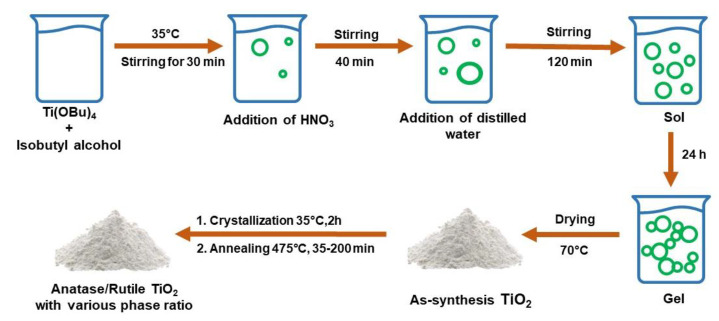
Schematic of sol–gel method to produce anatase/rutile heterophase.

In general, the sol–gel process involves a system transition from a liquid ‘sol’, which is mostly in the colloidal form, to a solid ‘gel’ phase. The starting materials used in the manufacture of ‘sol’ in the synthesis of TiO_2_ are usually metal alkoxide compounds such as titanium tetraisopropoxide (TTIP) and inorganic metal salts such as TiCl_4_ [210,211,212]. Arnal et al. [213] prepared titania by the sol–gel method from TiCl_4_ in diethyl ether, at 110 °C, to produce anatase. Meanwhile, for TiC1_4_ with ethanol, it leads to rutile as early as 110 °C, whereas the reaction of tert-butyl alcohol at 110 °C leads to the formation of a single brookite. The TiO_2_ polymorph heterophase synthesized by Castrejón-Sánchez et al. [214] which can control the anatase/rutile ratio. Initially, anatase-amorphous TiO_2_ powder was synthesized by the sol–gel method. Then, annealing was carried out with a time ranging from 35 to 200 min at 475 °C. By adjusting the annealing duration, it is feasible to manage the anatase/rutile ratio in nanostructured TiO_2_ powders from pure anatase to rutile. Figure 10 shows the sol–gel synthesis process with Ti(Obu)_4_ precursor using isobutyl alcohol and the addition of HNO_3_ to produce anatase/rutile heterophase.

#### 3.3.2. Hydrothermal Method

The hydrothermal process has strengths over other procedures, including excellent purity and crystallinity in the synthesized materials. Additionally, this technique yields a homogenous particle size distribution with a performance of more than 90% [215,216,217,218,219]. In the hydrothermal method, the trigger for the appearance of the rutile phase is pH [220], using the precursor [221], and using certain solvents [222]. The addition of additives can also trigger the appearance of the rutile phase in TiO_2_, such as adding urea as a nitrogen source in g-C_3_N_4_/TiO_2_ [222], tartaric acid (C_4_H_6_O_6_) [223], or adding metal impurities [224,225,226].

**Figure 11:**
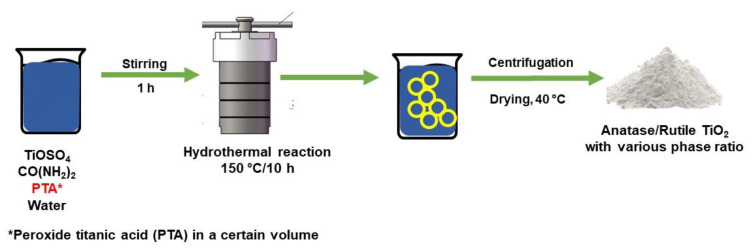
Schematic of hydrothermal method to produce anatase/rutile heterophase.

In a study conducted by Wang et al. [227], mixed-phase TiO_2_ was prepared by the hydrothermal method from TiOSO_4_ and peroxide titanic acid (PTA). The findings demonstrated that anatase and rutile phases made up the mixed-phase TiO_2_ powder, and that the PTA sol was crucial to the development of the rutile core. By adjusting the quantity of PTA sol utilized in the synthesis process, it is simple to control the amount of rutile in the TiO_2_ mixed phase between 0% and 70.5%. Figure 11 shows the hydrothermal method with addition of PTA to produce anatase/rutile heterophase. Meanwhile, in a study by Yang et al. [228], rutile, anatase, and brookite TiO_2_ nanorods were obtained by the hydrothermal method using PTA with different pH values. Rutile forms at pH values below 10, but anatase TiO_2_ can form at pH values over 10. Once the pH is 10, brookite can form.

#### 3.3.3. Sonochemical Method

**Figure 12:**
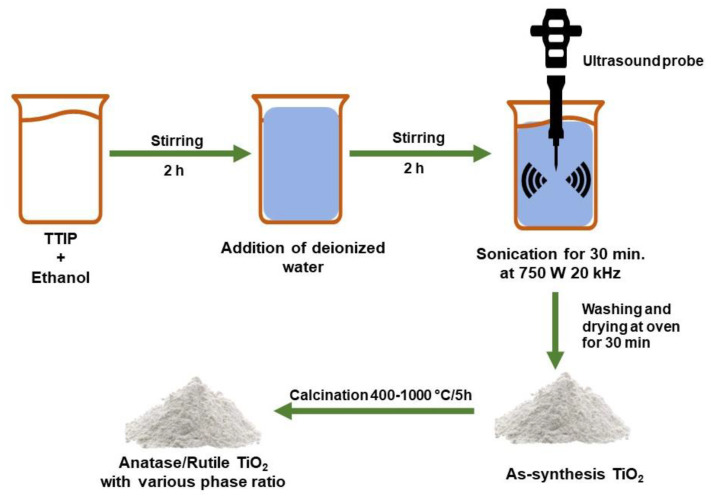
Schematic of sonochemical method to produce anatase/rutile heterophase.

Sonochemistry has been widely used to synthesize nano-sized materials [229,230,231,232,233]. Arami et al. [234] synthesized TiO_2_ nanoparticles with an average diameter of about 20 nm via a simple sonochemical method. The results indicated the nanoparticles consisted of a pure rutile phase. Meanwhile, for the mixed phase, anatase/rutile was prepared by a sonochemical process in which titanium(IV) isopropoxide (TTIP) was used as a precursor. The transformation of the anatase phase to the rutile phase in TiO_2_ powder was initially obtained by calcining the sample at 600 °C. The complete rutile phase occurs at the calcination temperature of 800 °C [235]. The best photocatalytic activity was demonstrated by an anatase/rutile composite with the proper ratio in a TiO_2_ photocatalyst that was calcined at 700 °C as a result of improved charge transfer at the mixed phase junction and reduced electron–hole pair recombination [236]. In addition, anatase/brookite composites have also been synthesized at 50 °C using sonochemical methods with similar crystal size and specific surface area, the photocatalytic activity of anatase/brookite for CH_3_CHO degradation is 5.4 times higher than in pure anatase. According to the electron energy loss spectrum, the collision of brookite and anatase crystals caused this high activity [237]. Figure 12 shows the sonochemical method with the TTIP precursor using ethanol and water as solvent to produce the anatase/rutile heterophase.

### 3.4. Transformation of Polymorph TiO_2_

The initial crystalline phase of TiO_2_ that forms during the various processes used to synthesize it is typically anatase. This may be owing to the usual TiO_6_ octahedral’s easier organization into a typical anatase structure when compared to rutile from a structural perspective [238]. From a thermodynamic perspective, the faster recrystallization of anatase can be due to the lower surface free energy compared to rutile [137]. However, some studies have been able to form rutile at room temperature conditions. The hydrothermal synthesis approach makes it possible to control the precipitation of rutile while facilitating the direct deposition of TiO_2_ crystals from the liquid phase. Rutile can only be obtained using this technique and high-temperature processing otherwise [239]. As the calcination changes in temperature, the proportions of rutile and anatase will progressively change as well. With higher calcination temperatures, the proportion of the rutile phase increases, which causes the proportion of the anatase phase to diminish [240]. Without any precursor or dopant modification, when making TiO_2_, the anatase to rutile conversion typically takes place between 600 and 700 °C [241]. Yuangpho et al. [242] examined the temperature of the anatase–rutile transformation and its impact on the material’s physical/chemical characteristics and photocatalytic activity. The photocatalytic activity of TiO_2_ decreases with increasing annealing temperature. These findings suggest that the phase composition influences the photocatalytic activity of TiO_2_ particles.

## 4. Photocatalytic Activity of Heterophase TiO_2_

### 4.1. Biphase of TiO_2_

#### 4.1.1. Anatase/Rutile

Anatase and rutile are the most common phase of TiO_2_ [243]. As a single phase, anatase has better photocatalytic activity than rutile [244]. However, the mixed anatase/rutile phases showed higher photocatalytic activity than single anatase [245]. Rutile is the most stable form compared to the other two forms that are metastable, brookite, and, anatase so at high temperatures it will turn into rutile [246]. It is common to regulate the calcination temperature to produce TiO_2_ with a mixture of anatase and rutile phases [247,248,249,250,251]. However, there are also other factors that can be used to control the appearance of these two phases such as the pH and the solvent used [252,253]. Several reports on the formation of mixed phase anatase/rutile structures are summarized in Table 2.

TiO_2_ with anatase/rutile can be used for the production of hydrogen, water splitting, and the degradation of organic pollutants. The factors that influence the increase in anatase/rutile photocatalyst activity are numerous energy-level staggered interfaces between the anatase and rutile, large specific surface area, and an enhancement in bridged hydroxyl functional groups on the surface [253].

Photocatalysis activity and degradation pathways from TiO_2_ with mixed phases are dependent on organic substances targeted by photocatalysis [254]. In many cases, increased anatase content tends to result in better TiO_2_ photocatalysis activity [255]. When combined with rutile, which is large in size and has high crystallinity, it will be an excellent photocatalytic. This is because degradation is a reaction that requires oxygen and anatase is a good oxygen absorber when compared to rutile. In addition, rutiles that have high crystallinity can also increase intrinsic photocatalysis activity [256]. The synergistic interaction between the anatase (011) and (110) rutile fields, which can also enhance the separation of electron holes and suppress electron and hole recombination, resulting in good photocatalysis activity [257]. Table 3 shows the performance of anatase/rutile photocatalytic degradation for the organic pollutants. Photocatalytic activity against the degradation of organic compounds is carried out by adding photocatalysts to the solution of organic compounds; then, concentration reduction was observed with a UV–vis spectrophotometer [247], or by measuring the conductivity of CO_2_ resulting from degradation [248]. For hydrogen generation, the hydrogen formation is measured with gas chromatography (GC) [249].

According to Ding et al. [63] the photocatalytic activity for the production of hydrogen (H_2_) and oxygen (O_2_) from pure water was significantly increased by the anatase/rutile synthesized using the hydrothermal/calcination method. It proves that rutile/anatase performed significantly better than pure rutile and pure anatase. In another study, anatase/rutile nanoparticles were modified with oxygen vacancies (TiO_2−x_). This heterophase TiO_2−x_ nanoparticles exhibit superior photocatalytic activity for converting CO_2_ to methane and can accelerate electron–hole separation [260].

#### 4.1.2. Anatase/Brookite

Among the three main crystallographic forms of TiO_2_, metastable brookite has received the least amount of research due to the difficulties with its synthesis in a pure form [261]. However, the development of several successful methods such as hydrothermal and sol–gel methods, pure brookite now exists. For photocatalytic activity, pure anatase exhibits a higher activity compared to other phases because the indirect band gap can exhibit lower recombination rates due to the longer lifetimes for photo-excited electrons and holes [70]. However, phase mixtures of various polymorphs are known to have synergistic effects and exhibit improved photocatalytic activity [56]. Compared to anatase/rutile, mixed-phase titania containing brookite has received less attention [262]. Table 4 summarizes a few studies on the preparation of mixed-phase TiO_2_ nanostructures with a tuned ratio of brookite in the titania mixtures. Meanwhile, Table 5 shows the performance of anatase/brookite photocatalytic degradation for the organic pollutants.

Anatase/brookite is successfully produced by hydrothermal method using titanium bis(ammonium lactate) dihydroxide (TALH) in the presence of high concentrations of urea (≥6.0 M) as an in situ OH− source. Lower urea concentrations lead to the formation of anatase/brookite mixtures. According to the results, pure anatase is less effective for hydrogen evolution than mixtures of anatase/brookite or pure brookite. On the other hand, pure brookite and mixtures of anatase and brookite show lower photocatalytic activity compared to pure anatase for the photocatalytic degradation of dichloroacetic acid (DCA) that performed at pH 3 [263]. However, TALH is neither affordable nor environmentally friendly (causing a release of ammonia) [262]. Another research work used titanium trichloride (TiCl_3_) as the titanium source, which can be easily manipulated, and uses tartaric acid (C_4_H_6_O_6_), which is low-cost and safe [265]. The as-prepared TiO_2_ that consisted of 78.7% anatase and 21.3% brookite showed the highest photocatalytic activity for Rhodamine B degradation, which was 1.2 times greater than commercial P25. A UV–Vis spectrometer was used to measure the maximum absorbance in order to determine the concentration of Rhodamine B. Meanwhile, anatase/brookite synthesized by an alkalescent hydrothermal treatment of TiCl_3_ by adjusting the NaCl concentration and NH_4_OH/H_2_O volume ratio showed that the product containing 53.4% brookite and 46.6% anatase shows the highest photocatalytic activity for degradation Rhodamine B, which is higher than pure phase [266]. For synthesized via a simple hydrothermal method with titanium sulfide (TiS_2_) as the precursors in sodium hydroxide solutions showed anatase/brookite TiO_2_ is 2.2 times more active—when measured by the H_2_ yield per unit area of the photocatalyst surface—than commercial P25 [267]. Another research work using titanium(III) sulfate (Ti_2_(SO_4_)_3_) in the presence of glycine through hydrothermal treatment shows that a sample containing 38.2% brookite and 61.8% anatase exhibited the highest efficiency for the removal cylindrospermopsin (CYN) and diclofenac than pure anatase, pure brookite, and commercial P25. The concentration of CYN was analyzed by high-performance liquid chromatography with a photodiode-array detector (PDA). Meanwhile, diclofenac concentration is measured by the UV–vis spectrophotometer [262,267].

TiO_2_ nanoparticles with anatase/brookite were also synthesized using a modified sol–gel method at low temperatures [268]. The mixture obtained at pH 2 and calcined at 200 °C had the highest activity for methylene blue degradation that measured by UV-Vis spectroscopy at 660 nm compared to commercial P25. The modified conventional sol–gel method is using the microwave-assisted sol–gel technique [269]. The phase transformation of TiO_2_ was investigated by hydrochloric and acetic acid. Three titania polymorphs were found when hydrochloric acid was used as a catalyst. On the other hand, a single anatase phase was obtained when acetic acid was added after only 15 min of reaction time. The ultrasonic-assisted sol–gel method [270], which used weak and strong acids, demonstrated a significant effect on their morphology, size, crystallinity, and photocatalytic performance. The photocatalytic activity showed that anatase and rutile phases with a high proportion of anatase (69:31 and 93:7, respectively), had the highest photoactivity for the degradation of MB compared with anatase/brookite (70:30).

According to Cihlar et al. [271], anatase/brookite is produced using the sol–gel complex method. This method consists of sol–gel, hydrothermal, and solid-state reactions that used titanium tetraisopropoxide (TTIP) as the precursor and the complex-forming (chelate-forming) substances used were glycine, EDTA, acetylacetone, and hydroxycarboxylic acids (lactic acid (LA), citric acid (CA), and tartaric acid (TA)) and their salts. These materials use to produce hydrogen by photocatalysis. The results show that anatase/brookite nanoparticles with 36% brookite, which were made using lactic acid, catalyzed the maximum rate of H_2_ evolution. The presence of anatase particles made using acetylacetone was found to have the lowest rate of H_2_ evolution. Another research work investigated the phase composition of TiO_2_ using lactic acid/Ti molar ratio ranging from 0.02 to 3.0 ratio via sol–gel complex synthesis [272]. Anatase/brookite was formed at low LA/Ti molar ratios, followed by anatase/brookite/rutile particles at average molar ratios, and pure anatase at high molar ratios. Anatase/brookite nanoparticles made at LA/Ti molar ratios between 0.03 and 0.1 showed the highest photocatalytic activity in the production of hydrogen by water splitting. Table 6 shows the performance of heterophase TiO_2_ for hydrogen production.

#### 4.1.3. Rutile/Brookite

Rutile/brookite as a photocatalyst was reported in 2008 by Di Paola et al. [273]. The sample was synthesized by thermohydrolysis of TiCl_4_ in HCl or NaCl aqueous solutions. Rutile mixtures were obtained depending on the acidity of the medium. The photocatalytic activity was evaluated by 4-nitrophenol degradation and quantitative determination was performed by measuring its absorption at 315 nm with a spectrophotometer UV. The highest photocatalytic activity for 4-nitrophenol degradation corresponded to the powders consisting of heterophase, compared to the pure phase [274].

Another way to synthesize a controlled rutile/brookite is by the hydrothermal method. This method uses titanium tetrachloride as a titanium source and triethylamine as a “regulating reagent” to adjust the ratio of brookite to rutile. The research found that the TiO_2_ sample with the highest photocatalytic activity for RhB degradation was obtained in a solution of 3 mL triethylamine and 10 mL water and contained 38% brookite and 62% rutile [275].

In another study, brookite/rutile was obtained using the solvothermal method. The morphology and structure of the samples were greatly changed by varying the ratio of TiCl_4_ to t-BuOH precursors. The phenol degradation measured by HPLC system showed that the highest photocatalytic activity was obtained from a composition of 72% brookite and 28% rutile. The highest phenol degradation activity was obtained from a composition of 72% brookite and 28% rutile. This indicates that the biphase of brookite/rutile with optimized phase proportions is responsible for the efficient synergy effect [276,277].

#### 4.1.4. Biphase of TiO_2_ (B)

TiO_2_ (B) is a less stable phase of TiO_2_ than anatase and rutile [114]. When compared to anatase, rutile, and brookite, TiO_2_ (B) is the least dense polymorph of TiO_2_ [278]. TiO_2_ (B) is suitable for use as a place of Li intercalation because it has a structure that is relatively open, with free space and continuous channels [115]. The synthetic TiO_2_ (B) nanowire-based electrode exhibited unique electronic properties, e.g., favourable charge-transfer ability, negative-shifted appeared flat-band potential, the existence of abundant surface states or oxygen vacancies, and high-level donor density [279,280]. There is an alternative synthesis of TiO_2_ (B), through the formation of a titanium complex obtained from the reaction between titanium metal powder and H_2_O_2_, NH_3_, and glycolic acid which produces a yellowish gel. The resulting gel is then added to H_2_SO_4_ to change the pH and put into the autoclave for hydrothermal treatment [173]. According to research by Wang et al. [73], the right quantity of HF could prevent the phase transition of TiO_2_ (B) to anatase. The mechanism of this phase transformation occurs because the F anion adsorbed on the surface of TiO_2_ (B) can efficiently reduce the surface energy from 0.63 J/m^2^ for a clean surface to −0.22 J/m^2^ for the surface adsorbed on F anion. Several methods for synthesizing TiO_2_ (B) are shown in Table 7.

However, TiO_2_ (B), as a photocatalyst composited with another TiO_2_ polymorph, was performed by Li et al. [67]. The biphase of TiO_2_ was synthesized from K_2_Ti_2_O_5_ through ion exchange and calcination. The nanofiber of core–shell crystal structure had a thin TiO_2_ (B) shell and an anatase core. The core–shell anatase/TiO_2_ (B) nanofiber showed increased photocatalytic activity in iodine oxidation reactions, with a reaction increase of 20–50% compared to single crystal anatase nanofiber or single crystal TiO_2_ (B). The same m was also conducted by Yang et al. [61], by synthesizing core–shell TiO_2_ (B)/anatase. A number of characteristics of this unusual structure improve the photocatalytic activity against the degradation of sulforhodamine B when exposed to UV light.

**Figure 13:**
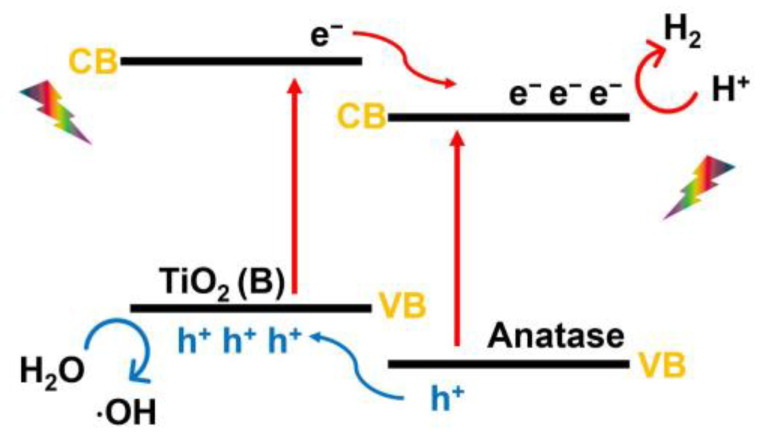
Schematic diagram illustrating the charge transfer across the TiO_2_ (B)/anatase heterophase junction.

The advantage of having a biphase of TiO_2_ (B)/anatase is that TiO_2_ (B) and anatase will form a heterojunction; the photogenerated electrons and holes can be transferred to the anatase phase and TiO_2_ (B) phase, respectively (Figure 13), thereby reducing recombination [195,284]. In the study of Mikrut et al. [285], TiO_2_ (B)/anatase was synthesized in the form of nanobelts. Similar to the anatase/rutile composite, a synergistic effect of the presence of the two phases was observed for TiO_2_ (B)/anatase (2:98). It is well known that TiO_2_ (B) is regarded as an optimized photocatalyst component for oxidation reactions involving hydroxyl radicals rather than promoting hydrogen evolution reactions.

**Figure 14:**
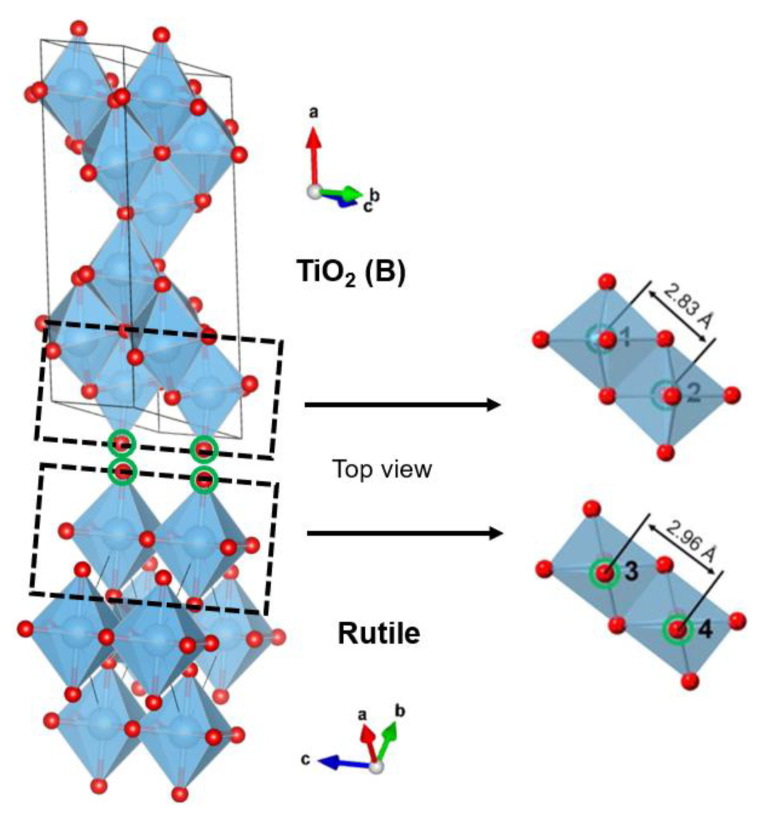
Possible connection model of the mixed phase at the phase interface.

Zhu et al. [286] reported another TiO_2_ (B) heterophase. In their research, the disintegration of TiO_2_ (B)/rutile used hydrothermal and calcination methods. The two phases are connected by an angle division at the phase interface (Figure 14). Electrons and holes are effectively separated across the phase interface as a result of the different conduction band and valence band positions between the two phases. The best results as photocatalysis were obtained at a TiO_2_ (B)/rutile ratio of 2/1, indicating the highest photocurrent and the best H_2_ evolution performance.

### 4.2. Triphase of TiO_2_

Numerous studies have been conducted to improve the activity of TiO_2_, but the two most significant findings are increasing the specific surface area and using the mixed phase [287]. The triphase of polymorph TiO_2_ shows a significant increase in photocatalytic activity compared to the pure and biphase phases [71]. Allen et al. [288] reported the effect of thermal treatment of heterophase polymorph TiO_2_ prepared by hydrolyzing titanium tetraisopropoxide at room temperature, dried at 382 K, and calcined at different temperatures for 1 h up to 1172 K. The results demonstrate that a mixture of brookite and anatase phases were seen up to 772 K, while a mixture of all three phases (anatase, brookite, and rutile) was present at 872 K, and a rutile-only phase was present at 1097 K and above.

Fischer et al. [70], synthesized a mixture of TiO_2_ phases (anatase, rutile, and brookite) on a polyethersulfone (PES) membrane via low-temperature dissolution-precipitation. In order to achieve that, the concentration of titanium precursor (titanium(IV) isopropoxide) was held constant while the amounts of hydrochloric acid and reaction temperature were adjusted to range from 0.1 to 1 M and 25 to 130 °C, respectively. The result showed that the best degradation rate of methylene blue were measured via microplate reader at a wavelength of λ = 660 nm, showing the highest activity that was obtained by 79% anatase and 21% brookite. Meanwhile, the recovery test showed that anatase, brookite, and rutile samples (70, 26, and 4%, respectively) did not degrade and completely recovered their photocatalytic abilities after at least nine additional cycles.

In a different study by Kaplan et al. [289], the formation of TiO_2_ that consists of anatase (43%), rutile (24%), and brookite (33%) was obtained by synthesizing TiO_2_ mixed phase using the sol–gel method and then hydrothermally treating it at a mild temperature (175 °C, 24 h). It was discovered that the photocatalytic activity of TiO_2_ nanocomposite successfully converted nearly 60% of the pollutant bisphenol into CO_2_ in H_2_O after being irradiated by UV light for 60 min. In contrast, only a lower level of mineralization was attained by the TiO_2_ P25 Degussa benchmark catalyst. This is due to a significantly reduced resistance to the accumulation of carbonaceous deposits on the catalyst surface.

## 5. Conclusions

This review summarizes most of the papers on TiO_2_ polymorphs (anatase, rutile, brookite, and TiO_2_ (B)), pure-phase TiO_2_ photocatalytic mechanisms, mixed phase, synthesis methods, and photocatalytic activities. This paper explains how to obtain pure phases from various TiO_2_ polymorphs and examples of their application in photocatalysis. In many cases, anatase has better activity than rutile and brookite. However, several studies have shown that rutile and brookite may have better activity in certain cases. Even thus, one-phase TiO_2_ has some drawbacks, such as high recombination and low specific surface area. TiO_2_ mixed phase shows better activity than the pure phase. This is due to good charge separation due to the formation of junctions around the interface. In addition, the synthesis methods that are often used to prepare mixed-phase TiO_2_ are sol–gel, hydrothermal, and sonochemical methods. Although the phase controller is usually the annealing temperature for anatase/rutile, in the hydrothermal method, additives are usually used as phase controllers.

Photocatalytic activity shows that anatase/rutile has much better activity than pure anatase or rutile. In addition, development continues to see the activity of other heterophases, such as anatase/brookite, rutile/brookite, and the heterophase TiO_2_ (B). However, in the case of brookite and TiO_2_ (B), the synthesis tends to be more difficult, these heterophase results show good photocatalytic activity. In addition, the triphase of polymorph TiO_2_ showed good photocatalytic activities for organic pollutants degradation. However, further studies to determine the exact number of compositions for each polymorph for the best activity in triphase TiO_2_ have not yet been investigated. In conclusion, further investigations are recommended to explore an easy method to synthesize TiO_2_ polymorphs with a high specific surface area and investigate the best composition of each TiO_2_ polymorph for photocatalytic activity, especially for triphase.

## Figures and Tables

**Table 1:** Crystal structure and physical parameters of TiO_2_ polymorphs.

Phase	Crystal System, Space Group	Lattice Parameter	Density (g/cm^3^)	Band Gap Energy (eV)	Ref.
Brookite	Ortorombik, *Pbca*	*a* = 9.148 Å	4.12	3.14–3.31	[117]
		*b* = 5.447 Å			
		*c* = 5.145 Å			
		*V* = 257.38 Å^3^			
Rutile	Tetragonal, *P*4_2_/*mnm*	*a* = *b* = 4.594 Å	4.25	3.02–3.04	[117]
		*c* = 2.959 Å			
		*V* = 62.45 Å^3^			
Anatase	Tetragonal, *I*4_1_/*amd*	*a* = *b* = 3.784 Å	3.89	3.20–3.23	[117]
		*c* = 9.515 Å			
		*V* = 136.24 Å^3^			
TiO_2_ (B)	Monoclinic, *C*2/*m*	*a* = 12.179 Å	3.73	3.09–3.22	[113]
		*b* = 3.741 Å			
		*c* = 6.525 Å			
		*β* = 107.054			
		*V* = 284.22 Å^3^			

**Table 2:** Various methods for synthesis TiO_2_ anatase/rutile.

Precursor	Method	Phase Controller	Application	Ref.
TiO_2_ P25	Hydrothermal	Calcination temperature	Water splitting	[63]
TTIP	Hydrothermal	Calcination temperature	Methylene blue degradation	[247]
TTIP	Sol–gel	Calcination temperature	Oxalic acid degradation	[248]
TBOT	Hydrothermal	pH	Hydrogen generation	[252]
TiOSO_4_	Urea precipitation method	Calcination temperature	4-chlorophenol degradation	[250]
TiCl_4_	One step condensing reflux	Solvent	Rhodamine B degradation	[253]
TiCl_3_	Hydrothermal	Calcination temperature	Hydrogen generation	[251]

**Table 3:** Photocatalytic activity of heterophase anatase/rutile.

Pollutant	Optimum Composition (%) *	Dye Concentration	Light Source	Irradiation Time (min)	Efficiency (%)	Ref.
Methylene blue	A 78.5 R 21.5	1 × 10^−5^ M	UV lamp	20	~94%	[248]
Rhodamine B	A 48.0 R 52.0	4.8 mg/L	300 W Xenon lamp (365 nm)	60	99%	[253]
Methyl orange	A 70.0 R 30.0	20 mg/L	125 W Mercury lamp (365 nm)	40	90.6%	[258]
Methylene blue	A 30.8 R 69.2	2.92 × 10^−5^ M	175 W UV lamp	90	85.8%	[220]
Crystal violet	A 81.6 R 18.4	100 ppm	UV lamp (365 nm)	300	92.65%	[259]
Methylene blue	A 81.6 R 18.4	100 ppm	UV lamp (365 nm)	300	94.77%	[259]

* A: Anatase, R: Rutile.

**Table 4:** Various methods for synthesis TiO_2_ anatase/brookite.

Precursor	Method	Additive	Application	Ref.
TALH	Hydrothermal	Urea	Hydrogen production and dichloroacetic acid (DCA) degradation	[263]
TiCl_3_	Hydrothermal	Tartaric acid	Rhodamine B degradation	[264]
TiCl_3_	Hydrothermal	NaCl and NH_4_OH	Rhodamine B degradation	[265]
TiS_2_	Hydrothermal	NaOH	Hydrogen generation	[266]
Ti_2_(SO_4_)_3_	Hydrothermal	Glycine with NH_4_OH or NaOH	Cylindrospermopsin degradation	[267]
TTIP	Sol–gel	HNO_3_	Methylene blue degradation	[268]
TTIP	Microwave assisted sol–gel	HCl and CH_3_COOH	-	[269]
TTIP	Ultrasonic-assisted sol–gel	HNO_3_ and CH_3_COOH	Methylene blue degradation	[270]
TTIP	Sol–gel complex	hydroxycarboxylic acids	Hydrogen generation	[271]
Ti(Opr)_4_	Sol–gel complex	Lactic acid	Hydrogen generation	[272]
Ti_2_(SO_4_)_3_	Hydrothermal	Glycine with NH_4_OH or NaOH	Diclofenac degradation	[273]

**Table 5:** Photocatalytic activity of heterophase anatase/brookite.

Pollutant	Optimum Composition (%) *	Photocatalyst Loading	Dye Concentration	Light Source	Irradiation Time (min)	Efficiency (%)	Ref.
Rhodamine B	A 78.7 B 21.3	-	20 mg/L	300 W Hg lamp (365 nm)	120	98	[264]
Rhodamine B	A 46.6 B 53.4	-	20 mg/L	300 W Hg lamp (365 nm)	100	95	[265]
Methylene blue	A 80.0 B 20.0	0.6 g/L	32 mg/L	100 W mercury lamp	70	98	[268]
Methylene blue	A 79.0 B 21.0	1 g/L	5 mg/L	100 W UV lamp (365 nm)	240	60	[270]
Cylindrospermopsin (CYN)	A 61.8 B 38.2	0.25 g/L	1 × 10^−6^ M	15 W florescence lamp (310–720 nm)	15	100	[267]
Diclofenac (DCF)	A 61.8 B 38.2	1 g/L	10 mg/L	UV-A irradiation	120	100	[262]

* A: Anatase, B: Brookite.

**Table 6:** Photocatalytic activity of heterophase TiO_2_ for hydrogen production.

Optimum Composition (%) *	Amount of Catalyst	Light Source	H_2_ Evolution Rate	Ref.
A 73.5 R 26.5	20 mg/100 mL	300 W Xe lamp	584 μmol g^−1^ h^−1^	[63]
A 12.0 R 88.0	20 mg/80 mL	350 W Xe lamp	74,400 μmol g^−1^ h^−1^	[252]
A 72.0 B 28.0	37.5 mg/75 mL	1000 W Xe lamp	4267 μmol g^−1^ h^−1^	[263]
A 88.4 B 11.6	45 mg/100 mL	800 W Xe−Hg lamp	~3500 μmol g^−1^ h^−1^	[266]
A 74.0 B 36.0	20 mg/100 mL	450 W Xe lamp	101.4 μmol g^−1^ min^−1^	[271]
A 54.0 B 46.0	20 mg/100 mL	450 W Xe lamp	101.4 μmol g^−1^ min^−1^	[272]

* A: anatase, R: rutile, B: brookite.

**Table 7:** Various methods for synthesis TiO_2_ (B).

Precursor	Method	Additive	Application	Ref.
Titanium metal powder	Post-synthetic Hydrothermal crystal growth	H_2_SO_4_	-	[281]
Titanium metal powder	Hydrothermal	H_2_SO_4_	-	[282]
TiCl_4_	Solvothermal	-	Lithium-ion batteries	[173]
TiO_2_ P25	Hydrothermal	-	DSSC	[278]
TiO_2_ P25	Hydrothermal	-	Humidity sensors	[279]
TTIP	Surfactant-assisted nonaqueous sol–gel route	Oleic acid	Lithium ion-batteries	[283]

## Data Availability

The study did not report any data.
